# How to proceed when evidence-based practice is required but very little evidence available?

**DOI:** 10.1186/2045-709X-21-24

**Published:** 2013-07-10

**Authors:** Charlotte Leboeuf-Yde, Olivier Lanlo, Bruce F Walker

**Affiliations:** 1 The Spine Research Centre, Hospital Lillebaelt, and Institute for Regional Health Research, University of Southern Denmark, Middelfart, Denmark; 2 Complexité, Innovation et Activités Motrices et Sportives, Bâtiment 335, UFR STAPS, Université Paris Sud-11, Orsay Cedex 91405, France; 3 Institut Franco-Européen de Chiropraxie, Paris, France; 4 School of Health Professions, Murdoch University, Perth, Western Australia, Australia

**Keywords:** Chiropractic, Evidence-based practice, Biological plausibility

## Abstract

**Background:**

All clinicians of today know that scientific evidence is the base on which clinical practice should rest. However, this is not always easy, in particular in those disciplines, where the evidence is scarce. Although the last decades have brought an impressive production of research that is of interest to chiropractors, there are still many areas such as diagnosis, prognosis, choice of treatment, and management that have not been subjected to extensive scrutiny.

**Discussion:**

In this paper we argue that a simple system consisting of three questions will help clinicians deal with some of the complexities of clinical practice, in particular what to do when clear clinical evidence is lacking. Question 1 asks: are there objectively tested facts to support the concept? Question 2: are the concepts that form the basis for this clinical act or decision based on scientifically acceptable concepts? And question three; is the concept based on long-term and widely accepted experience? This method that we call the *“Traffic Light System”* can be applied to most clinical processes.

**Summary:**

We explain how the Traffic Light System can be used as a simple framework to help chiropractors make clinical decisions in a simple and lucid manner. We do this by explaining the roles of biological plausibility and clinical experience and how they should be weighted in relation to scientific evidence in the clinical decision making process, and in particular how to proceed, when evidence is missing.

## Background

### The clinical encounter has many aspects – evidence-based practice expected

For health care practitioners, the first clinical encounter has several important goals, two of these are: 1) to get an idea of what is wrong with the patient and 2) to detect any cases that should be directed elsewhere. Chiropractors, and other professionals who often provide manual therapies, will also work out a “technical” diagnosis in relation to where and what to treat. Thereafter follows the treatment itself, any follow-up procedure, and the long-term strategy. This is a relatively complex process, as many different pieces of information must be considered, brought into a coherent picture and acted upon. In addition, clinicians are expected to keep updated on that part of dynamic knowledge – research – that concerns their area of practice and to apply this knowledge on each patient.

All clinicians of today know that scientific evidence is the base on which clinical practice should rest. However, this is not always easy, in particular in those disciplines, where the evidence is scarce. There is also the issue of the definition of “evidence”. Textbooks have been devoted to this. Throughout this text we shall assume that “evidence” equals the “best evidence” available at the time, when evaluating the value of a clinical procedure.

Although the last decades have brought an impressive production of research that is of interest to chiropractors, there are still many areas such as diagnosis, prognosis, choice of treatment, and management that have not been subjected to extensive scrutiny. Therefore, some aspects of health care are accepted on their logic and face value and through their repeated and successful use over time.

### If no evidence: plausibility and experience

This lack of specific evidence, however, is not always worrying, as it is considered acceptable to extrapolate from generally acknowledged scientific concepts, and it follows that procedures and concepts will, generally, not be questioned when this happens.

If the underlying rationale is plausible, in addition to the procedure or concept being accepted by many clinicians, then it is likely that it will be considered relatively “acceptable”. However, the level of acceptance is lower for only logic and experience than it is for scientific evidence. This means that decisions and actions based solely on logic and experience usually cannot stand on their own. An example is that the plausible and frequently used test for an acutely injured lumbar disc, antalgia on flexion of the lumbar spine, can be used to help diagnose this disc problem [1], but that other corroborating information is needed before the diagnosis is accepted.

### Lack of generally acceptable logical concept

However, if the concept, act or procedure is not biologically plausible, as judged by the scientific community in general, then experience is not enough to justify its use. This is not surprising, because if a procedure or decision is based on a biologically implausible rationale, it is unlikely that it will be clinically valid.

An example of a non-acceptable procedure is the use of a pendulum in order to define the gender of the unborn baby. This method lacks an acceptable contemporary scientific rationale, so even if some people think that this method is useful, it cannot be introduced in an obstetrics department, unless several well carried out studies have shown, unequivocally, that the vast majority of unborn babies can be correctly classified by gender using a pendulum. Thus a very heavy onus of proof would rest upon any person who would claim that this method can be used for this purpose.

On the other hand, if, against all odds, a test or treatment that lacks (contemporary) plausibility is shown to be clinically valid in several well designed and appropriately performed studies, then it will be considered “acceptable”. The reason for this is, that the contemporary knowledge may not be sufficient to explain why this is so. An historical example of how, sadly, “the evidence” won over common sense and repeated observations is how dirty hands were dismissed as a cause of puerperal fever in childbirth, because its pathogens had not been discovered [2].

Sometimes the question on whether a concept is logical or not will be answered differently by different groups of practitioners. It is, however, outside the scope of this article to define further “logical” and “biologically plausible” in relation to various chiropractic concepts; this discussion belongs elsewhere. However, the plausibility here refers to that which would be acknowledged as such by the contemporary general scientific community.

### Easy clinical decisions vs. difficult decisions

Obviously, there are many aspects in clinical practice that lurk in the areas of no specific evidence yet considered to be perfectly acceptable because they are based on sound and generally accepted biological/physiological/anatomical/pathological concepts combined with longstanding and widespread experience.

For example within spinal care it would be considered acceptable to advise a patient with a very painful and antalgic lumbar spine against digging up his garden whilst in such a state. The rationale for this advice is that such activity would be likely to aggravate the affected spinal structures. This advice has never been tested in a randomized controlled clinical trial; instead it is based on our present understanding of the pathology of the disc and backed up by our experience of outcome in relation to whether this type of patients avoid aggravating activities or not.

However, there are other times when clinicians may become confused. For example, when the above patient asks how many treatments he will need and how frequently these should be administered, the rationale for this is not so clear. Should there be frequent treatments over a short period of time (rationale: the more the better) or does it suffice with few treatments (rationale: a few treatments will help the process on its way and healing takes the time it takes)? Further, the experiences of various clinicians may be difficult to evaluate, as each of them probably predominantly acknowledges their own specific rationale and therefore has limited experiences to draw on. Each is likely to assume that their specific approach and experiences represents the gold standard. We contend that the same uncertainty will arise over and over, as other unstudied cases present themselves in the practice.

### What to do in these cases?

It can be challenging and confusing to make clinical decisions in situations where clear evidence is lacking. Many people find it uncomfortable and difficult to deal with uncertainty and feel safer if they can follow an algorithm of thought, some sort of recipe on how to proceed. This probably explains the plethora of more or less complicated experience-based recipe-type techniques that are available in the chiropractic profession.

On the other hand, attempts have been made since the early 1990s to assist chiropractors to perform in an evidence-based and streamlined manner. Most manual and/or observational tests in many health care fields would probably be classified under this category. Examples are visual inspections of radiographs, orthopedic and neurological tests, and auscultation of the abdomen and heart. Other examples would be those chiropractic tests that have been poorly studied or studied with conflicting results yet based on a seemingly logical patho-anatomical concept.

Over the past 25 years, particular since the Mercy Guidelines [3] substantial efforts have resulted in several clinical algorithms pertinent to chiropractic practice in specific domains [4-7], but we have been unable to find a simplistic, general algorithm, applicable to chiropractic practice in general.

In this paper we argue that a simple system consisting of three questions will help clinicians deal with some of the complexities of clinical practice, in particular what to do when clear clinical evidence is lacking. This method, the *“Traffic Light System”*, can be applied to most clinical processes. We shall explain how the Traffic Light System can be used as a framework to help chiropractors make clinical decisions in a simple and lucid manner. We do this by explaining the roles of biological plausibility and clinical experience and how they should be weighted in relation to scientific evidence in the clinical decision making process.

**Figure 1:**
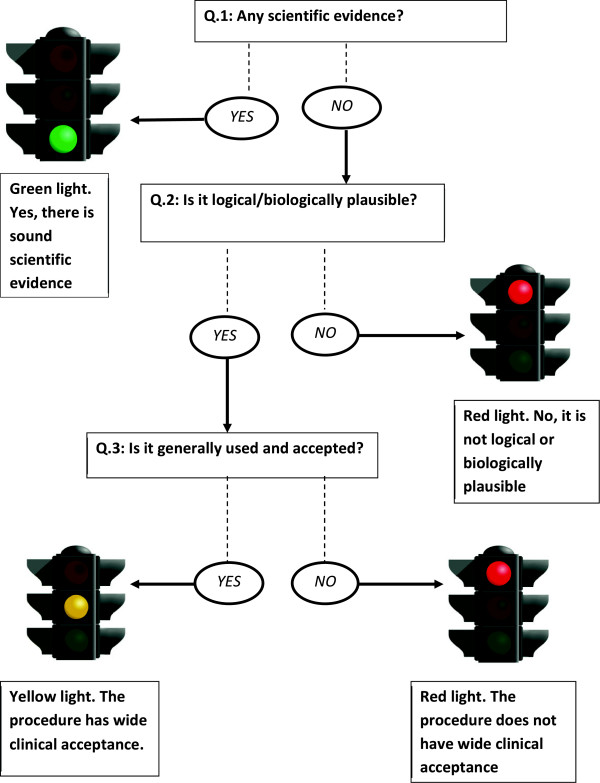
The Traffic Light System, a three-step algorithm for clinical reasoning in chiropractic practice.

According to the Traffic Light System, decision making is based on some simple concepts. First, scientific evidence carries more weight than biological plausibility and experience and, in particular, when there is lack of biological plausibility, there is a much stronger need for evidence. On the other hand, if there is biological plausibility, strong evidence may not always be needed, provided that the concept is also backed up by considerable clinical experience. This concept is illustrated in Figure 1.

We shall present two clinical situations in which the Traffic Light approach can be used: 1) when examining a patient, and 2) when choosing an appropriate method of treatment. However, we propose that the same approach can be used throughout the entire clinical decision-making process.

## Discussion

### The three questions in the traffic light system

The Traffic Light System is a method of approach that can be used to deal with the triad of: *evidence, plausibility and clinical experience*. We suggest that three questions be asked, whenever facing a clinical task, and that the continued activities should be based on a set method to interpret the answers to these questions. In order to provide a simple and easy-to-remember algorithm, we have incorporated the three traffic light colors into the system, green (go), yellow (go with care), and red (stop). The three questions are presented below together with their traffic light interpretations in three steps:

Step 1: Are there objectively tested facts to support the concept? In other words, is there sufficient sound research (evidence) supporting this? If the answer is “yes”, the light is *green,* and you can go on with your procedure or decision. If the answer is “no”, go to the second question.

Step 2: Are the concepts that form the basis for this clinical act or decision based on scientifically acceptable concepts? In other words, is it logical and lucid, biologically plausible? If the answer is “yes”, go to question three. If the answer is “no”, the light is *red* and you should stop here.

Step 3: Is the concept based on long-term and widely accepted experience? In other words, is ‘everybody’ doing it? If also this answer is “yes”, the light is *yellow* and you can proceed with care. If the answer is “no”, then stop for the *red* light.

The use of these three questions will now be demonstrated for two specific clinical aspects, 1) in the examination of the patient, and 2) in the choice of method of treatment. Depending on the answers to these questions, the case will be categorized with one of the three Traffic Light System colors (red, green or yellow).

### Two examples on how to apply the Traffic Light System

Example 1: Examining the patient

All questions, observations, and clinical examination procedures that are performed in order to obtain an answer to a clinical question can be classified as “tests”. Without discussing details, in general, tests should be used only if they are in some way useful, i.e. if they can help obtain a diagnosis, direct the type of treatment or hint to the prognosis. Tests that provide no such messages are a waste of time, and are to be considered meaningless rituals that may even confuse the issue. The only exception to this would be tests that are used to describe status over the course of treatment to see how the case progresses but play no role in the diagnosis, treatment or initial indications of prognosis. For example, information on particular positions (e.g. bending forward) or activities (e.g. turn in bed) which are painful.

We propose that these are *red light* tests:

1) The test has been submitted to scientific study and found not to “work” (step 1). This is regardless of whether it can be considered to be a plausible test (step 2) or if it is frequently used (step 3); we suggest that this is not a test to be used. In other words, if the test has been shown to be useless, it is not relevant to proceed to steps 2 and 3.

2) The test is illogical (step 2) and it has not been submitted to scientific study to prove its value (step 1). Because it is an illogical test, it would be necessary to have positive evidence to make this test acceptable. Therefore, it is not relevant to continue to step 3.

Some tests that make up the examination procedure of various chiropractic technique “systems” belong to this category.

Using the same approach, we propose that the following cases are considered to be *yellow light* tests:

1) The test has not been submitted to scientific scrutiny (step 1) but it is a logical test (step 2) that is generally found to be useful (step 3).

2) The test, which is logical (step 2), has been studied scientifically with differing results or with only moderate success (e.g.low to moderate sensitivity and/or specificity, or low to moderate reliability) (step 1).

Most manual and/or observational tests in many health care fields would probably be classified under this category of insufficient evidence. Examples are visual inspections of radiographs, orthopedic and neurological tests, and auscultation of the abdomen and heart. Another example would be the reliability and validity of the identification of a vertebral segment requiring manipulation by using, for example, various types of palpation; generally incompletely studied and/or with conflicting research results but for many seemingly logical.

According to our method of approach, *green light* tests are the following:

1) All tests that have been studied scientifically and found to work (step1), regardless of whether they are plausible or not (step 2).

2) Several moderately useful tests that, when combined, are found to ‘work’ (step 1).

Manual and observation tests in the green category are uncommon.

To conclude, illogical tests must be shown to be valid before they can be used, whereas logical and commonly used but ‘untested’ tests are relatively acceptable provided that major decisions are not based entirely on one such single test.

Example 2: Selecting the treatment

Once patients who are unsuitable for treatment have been excluded (because they have either a contra-indication or a non-indication to treatment), and patients who are thought suitable for treatment have been accepted (usually with a tentative diagnosis), the next question to be dealt with is: How should this patient be treated?

Again, we suggest that the Traffic Light System can be used in relation to the choice of treatment method. We shall assume that the chiropractor wishes to select a particular manual technique, either one that includes an analytic method (e.g. sacro-occipital technique [8]) or one that consists of a therapeutic method that stands on its own without the necessity of a special pre-determined analytic approach.

Using the same theoretical approach as previously, *red light* techniques are those that are 1) illogical and untested or 2) logical but tested and found not to “work”. *Yellow light* techniques are logical and used extensively with seemingly good results but “unproven” or with research findings indicating only moderately good results or with fluctuating study results between studies. *Green light* techniques are logical (or illogical), tested and found to “work”.

“Ordinary” generic manipulation has been used in randomized clinical trials in which the value of spinal manipulation was studied. The results of these studies generally indicate that there is some clinical effect albeit small and not much different to other modalities including sham [9]. So it could be concluded that spinal manipulation is ineffective. However, these trials usually direct the manipulation at a symptom i.e. non-specific back pain and not a diagnosis. Accordingly, it is possible that future research may establish a role for back manipulation for a subset of patients with pain amenable to this type of treatment. So what do we do in the meantime while we await further research? We believe that “ordinary” manipulation for back pain satisfies step 2 and 3 and can be proceeded with as a “Yellow light therapy”. However, it should be noted that there is presently scarcity of data on which type of technique (if any) is best for specific cases.

In the case where a treatment technique is encapsulated within a “system” such as the Sacro-Occipital Technique, it would be relevant to separate the analytical (diagnostic) part from the therapeutic part. The reason for this is that it would be possible for the analytical part (e.g. the body sway test, as performed in this technique) to be a “red light” test whilst the therapeutic aspect of the technique (for example pelvic blocking) could be yellow or even potentially green.

#### Summary

With these explanations we hope to have shown how the Traffic Light System can be used to guide chiropractors through a simple thought process, in which they should habitually consider three questions: Is there specific sound research evidence in favor of this clinical procedure? If not, is it at least logical/biologically plausible? And if so, has it gained wide clinical acceptance? Positive or negative answers to these questions will then be interpreted as one of the three lights, red, yellow or green. Green light means “go ahead”, yellow means “proceed with care”, whereas red means “stop”.

After having applied this method for a while, it will become clear that most clinical procedures in chiropractic practice (diagnostic tests and choice of specific techniques) probably belong under the yellow light.

This abundance of yellow lights makes it obvious that, although the “science of chiropractic practice” is very important, what the clinician often has to deal with is the “art of chiropractic”, in the general absence of evidence. This demands an ability to deal with uncertainty. How well this can be handled would depend on patient values and circumstances, but the practitioner’s own clinical experience and his/her own ethical stamina are also very important. These additional issues, therefore, deserve careful consideration.

## Competing interests

The authors declare that they have no competing interests.

## Authors’ contributions

CLY presented the concept and all authors discussed this, CLY drafted the manuscript, OL performed a literature review, and all authors reviewed, revised and accepted the final version. The concept has been previously partially presented at professional conferences. All authors read and approved the final manuscript.

## Authors’ information

Professor Charlotte Leboeuf-Yde, DC, MPH, PhD. The Spine Research Centre, Hospital Lillebaelt, and Institute for Regional Health Research, University of Southern Denmark, Middelfart, Denmark, and Visiting Professor, Complexité, Innovation et Activités Motrices et Sportives, Bâtiment 335, UFR STAPS, Université Paris Sud-11, Orsay Cedex 91405, France

Olivier Lanlo, DC, LLM, Executive President Institut Franco-Européen de Chiropraxie, Paris and Toulouse, France

Associate Professor Bruce Walker, School of Health Professions, Discipline of Chiropractic, Murdoch University, Perth, Western Australia, Australia

## Funding

Charlotte Leboeuf-Yde’s position in Denmark was until the 31.12.2012 partially funded by’Fonden til fremme for kiropraktisk forskning og postgraduat uddannelse’.
